# Analyzing Patients' Values by Applying Cluster Analysis and LRFM Model in a Pediatric Dental Clinic in Taiwan

**DOI:** 10.1155/2014/685495

**Published:** 2014-06-19

**Authors:** Hsin-Hung Wu, Shih-Yen Lin, Chih-Wei Liu

**Affiliations:** ^1^Department of Business Administration, National Changhua University of Education, Changhua City 500, Taiwan; ^2^Department of Tourism, Leisure, and Hospitality Management, National Chi Nan University, Nantou 545, Taiwan

## Abstract

This study combines cluster analysis and LRFM (length, recency, frequency, and monetary) model in a pediatric dental clinic in Taiwan to analyze patients' values. A two-stage approach by self-organizing maps and *K*-means method is applied to segment 1,462 patients into twelve clusters. The average values of *L*, *R*, and *F* excluding monetary covered by national health insurance program are computed for each cluster. In addition, customer value matrix is used to analyze customer values of twelve clusters in terms of frequency and monetary. Customer relationship matrix considering length and recency is also applied to classify different types of customers from these twelve clusters. The results show that three clusters can be classified into loyal patients with *L*, *R*, and *F* values greater than the respective average *L*, *R*, and *F* values, while three clusters can be viewed as lost patients without any variable above the average values of *L*, *R*, and *F*. When different types of patients are identified, marketing strategies can be designed to meet different patients' needs.

## 1. Introduction

The medical care industry in Taiwan has become very competitive when the national health insurance (NHI) program with a mandatory, single-payer social health insurance system was launched by the government in Taiwan since March 1995 [1, 2]. Under such system, each citizen has equal access to health care services, health care of acceptable quality, comprehensive benefits, and convenient access to treatment with low premiums and health care expenditures [1]. That is, citizens are free to choose health care providers and medical institutions with low reimbursement [2, 3]. Dental clinic in Taiwan is covered as part of the benefit package in NHI program with the cost-containment mechanism and global budgeting systems and the income of a dental clinic is mainly from the copayment and registration fee per visit [4]. This scenario shows that it is critically important for dental clinics to not only identify profitable patients but also retain as many patients as possible under the current environment.

Market segmentation is very useful to identify different customer needs and then to provide the management to customize the products or services to fulfill the needs [5]. Data mining techniques, particularly cluster analysis, enable the management to divide all customers into appropriate number of clusters in accordance with the similarities of the customers [6]. In addition, the value for each cluster can be computed and evaluated such that the management can effectively deploy resources to satisfy different customer needs. In addition, RFM (recency, frequency, and monetary) and LRFM (length, recency, frequency, and monetary) models, where LRFM model further considers the relationship length between the organization and customers, are typically applied along with cluster analysis to analyze customer values [3, 7, 8]. Therefore, a combination of LRFM model and cluster analysis would be helpful to the management to identify critical important customers. Then, the management can design different marketing strategies to maximize customer values.

This study intends to combine data mining techniques (cluster analysis) and LRFM model in a pediatric dental clinic for market segmentation. When all patients are grouped into clusters, the characteristics for each cluster are to be observed and described. By observing the average values of* L*,* R*, and* F* values compared with the overall average values of* L*,* R*, and* F* values, different types of patients, such as loyal patients, potential patients, and lost patients, can be identified. In addition, in order to evaluate customer values, customer value matrix proposed by Marcus [9] by considering frequency and monetary and customer relationship matrix depicted by Chang and Tsay [10] by taking into account length and recency are to be illustrated such that the management can further divide all patients into different types of patients. Therefore, the management can design different marketing strategies to meet different types of patients' needs when patients' values are evaluated.

This paper is organized as follows.  Section 2 briefly reviews cluster analysis, RFM and LRFM models, and customer values in terms of customer value matrix and customer relationship matrix. A case of analyzing patients' values of a pediatric dental clinic in Taiwan is illustrated in Section 3. Finally, conclusions are drawn in Section 4.

## 2. Literature Review of Cluster Analysis, LRFM Model, and Customer Values

### 2.1. Cluster Analysis

Huang et al. [6] stated that data mining techniques particularly cluster analysis can be applied to divide all customers into appropriate number of clusters based on some similarities among these customers when the transactions of an organization become much larger in size. The philosophy of cluster analysis is to maximize the intraclass similarities and minimize the interclass similarities [11]. That is, clusters are formed such that objects within a cluster have high similarity compared with one another but are very dissimilar to objects in other clusters. Wei et al. [8] pointed out that self-organizing maps (SOM) and* K*-means method are commonly used for cluster analysis.

SOM, an unsupervised neural network method, uses clustering, visualization, and abstraction to solve problems and market screening [12–15]. The concept of SOM is to detect strong features in large data sets and then produce two-dimensional arrangement of neurons from a multidimensional space. Because the patterns in a high-dimensional input space are very complicated, SOM reduces the complexity by providing a projected graphical map display, which becomes more transparent and understandable [8, 16].

*K*-means method, a nonhierarchical approach, is one of the very popular approaches to perform cluster analysis due to its simplicity of implementation and fast execution [17].* K*-means method using Euclidean distance has two major steps. First, place the instances in the closest class (the assignment step). Second, recalculate class centroids from the instances assigned to the class (the reestimation step) [6, 17].

Self-organizing maps method has the advantages over other traditional cluster analysis methods but it does not provide measures for validation for the cluster analysis results [18]. In contrast,* K*-means method is very sensitive to the choice of a starting point to partition the items into* K* initial clusters [5, 19, 20]. Therefore, a two-stage approach combining SOM and* K*-means method has been proposed and widely applied to improve the weakness [8, 20, 21]. That is, the data set is clustered by SOM to determine the number of the clusters in the first stage. Later, the determined number of the clusters is used for* K*-means method to perform cluster analysis of the data set in the second stage.

### 2.2. LRFM Model

LRFM model was developed based on RFM model, which is a well-known method to analyze customer values for market segmentation [19, 22]. The definition of RFM model is as follows [23, 24]: recency is defined as the number of periods since the last purchase, that is, days or month. Frequency is the number of purchases made in a given time period. Monetary can be measured by the total amount of the money spent during a given time period or the average amount of money in the time period.

The traditional approach to use RFM model is to sort the customer data and then divide the data into five equal segments for each dimension of RFM [22]. The top 20% segment is assigned as a value of 5, the next 20% segment is assigned as a value of 4, and so on. Thus, each customer based on RFM model can be represented by one of 125 RFM cells, namely, 555, 554, 553, …, 111 [22, 25]. Chang et al. [19], on the other hand, use the original data rather than the coded number to perform RFM model. The definitions are as follows: recency is the time length since the most recent purchase; frequency computes the number of purchases during the same period of time; and monetary refers to the total amount of money spent on all purchases given the same period of time.

Reinartz and Kumar [26] addressed that RFM model cannot distinguish which customers have long-term or short-term relationships with the company. The customer loyalty depending on the relationship between a company and its customers is established from a long-term customer relationship management [10]. Therefore, Chang and Tsay [10] extended RFM model to LRFM model by taking length (*L*) into consideration, where* L* is defined as the number of time periods (such as days) from the first purchase to the last purchase in the database.

### 2.3. Customer Values

Ha and Park [27] used average RFM values of each cluster to compare with the total average RFM values of all clusters. If the average value is greater than the total average, an upward arrow (↑) is given to that particular symbol, while a downward arrow (↓) is provided when the average value is less than the total average. Thus, the combinations of RFM model can be classified into eight categories. In our study,* R* is redefined as the number of days since the last visit when the first day of the specified time period is set to one. That is,* R* value belongs to the larger-is-better scenario, and larger* R* value indicates that the customer has visited the organization more recently, which is different from the traditional definition of recency. By further taking into account the strategic positioning of customer clusters, four major types of customers can be grouped in terms of RFM symbols, that is, lost customers with  *R* **↓** *F* **↓** *M* **↓** and* R* **↓** *F* **↑** *M* **↑**, new customers with* R* **↑** *F* **↓** *M* **↓,** loyal customers with* R* **↑** *F* **↑** *M* **↑**, and promising customers with* R* **↑** *F* **↓** *M* **↑**.

Chang and Tsay [10] based on Ha and Park [27] defined four types of customers in terms of LRFM model, including best customers (*L* **↑** *R* **↑** *F* **↑** *M* **↑**,* L* **↓** *R* **↑** *F* **↑** *M* **↑**,* L* **↑** *R* **↓** *F* **↑** *M* **↑**, and* L* **↓** *R* **↓** *F* **↑** *M* **↑**), frequent customers (*L* **↑** *R* **↑** *F* **↑** *M* **↓**,* L* **↓** *R* **↑** *F* **↑** *M* **↓**,* L* **↑** *R* **↓** *F* **↑** *M* **↓**, and* L* **↓** *R* **↓** *F* **↑** *M* **↓**), spender customers (*L* **↑** *R* **↑** *F* **↓** *M* **↑**,* L* **↓** *R* **↑** *F* **↓** *M* **↑**,* L* **↑** *R* **↓** *F* **↓** *M* **↑**, and* L* **↓** *R* **↓** *F* **↓** *M* **↑**), and uncertain customers (*L* **↑** *R* **↑** *F* **↓** *M* **↓**,* L* **↓** *R* **↑** *F* **↓** *M* **↓**,* L* **↑** *R* **↓** *F* **↓** *M* **↓**, and* L* **↓** *R* **↓** *F* **↓** *M* **↓**). Moreover, sixteen combinations of LRFM model can be classified into five major groups such as core customers, potential customers, lost customers, new customers, and resource-consumption customers. Specifically, core customers include* L*↑*R*↑*F*↑*M*↑,* L*↑*R*↑*F*↑*M*↓, and* L*↑*R*↑*F*↓*M*↑. Potential customers consist of* L*↑*R*↓*F*↑*M*↑,* L*↑*R*↓*F*↑*M*↓, and* L*↑*R*↓*F*↓*M*↑. Lost customers are composed of* L*↓*R*↓*F*↑*M*↑,* L*↓*R*↓*F*↑*M*↓,* L*↓*R*↓*F*↓*M*↑, and* L*↓*R*↓*F*↓*M*↓. New customers comprise* L*↓*R*↑*F*↓*M*↓,* L*↓*R*↑*F*↑*M*↓,* L*↓*R*↑*F*↓*M*↑, and* L*↓*R*↑*F*↓*M*↓. Finally, resource-consumption customers are* L*↑*R*↑*F*↓*M*↓ and* L*↑*R*↓*F*↓*M*↓.

**Figure 1:**
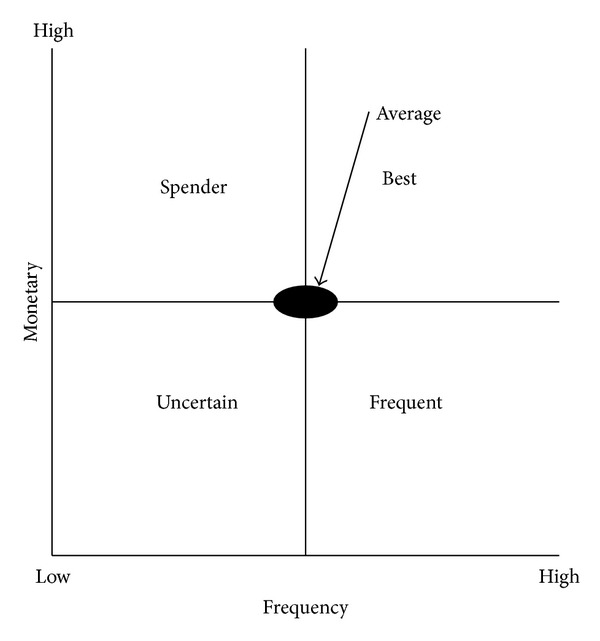
The customer value matrix.

**Figure 2:**
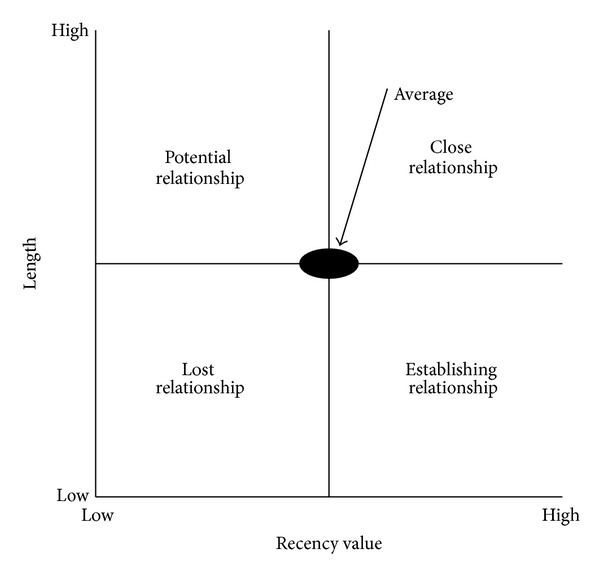
Customer relationship matrix.

Marcus [9] proposed the concept of the customer value matrix which is very suitable for small retail and service businesses to analyze customer values. By using the average* F* and* M* values, four quadrants as shown in Figure 1 are formed, including best customers (*F*↑*M*↑), spender customers (*F*↓*M*↑), uncertain customers (*F*↓*M*↓), and frequent customers (*F*↑*M*↓). On the other hand, Chang and Tsay [10] used* L* and* R* to explain the characteristics of lost customer and loyalty by proposing customer relationship matrix depicted in Figure 2 with close relationship (*L*↑*R*↑), potential relationship (*L*↑*R*↓), lost relationship (*L*↓*R*↓), and establishing relationship (*L*↓*R*↑).

When different LRFM combinations are identified, customers can be classified into appropriate groups such as core customers, potential customers, lost customers, new customers, and resource-consumption customers. In addition, the customer value matrix enables the management to classify customers into best, spender, uncertain, and frequent customers in terms of frequency and monetary. Customer relationship matrix helps the management identify the characteristics of four different types of the relationships between the organization and the customers through length and recency. In other words, customer values can be evaluated by customer value matrix and customer relationship matrix. More importantly, different marketing strategies can be designed to meet different customer needs.

## 3. A Case of Analyzing Patients' Values of a Pediatric Dental Clinic in Taiwan

This pediatric dental clinic started its operation since 17 September 1995. By definition, the patients must be less than 18 years old. This study collects the data set with 1,462 effective patients who visited this clinic with the total of 7,957 times from 1 July 2009 to 30 June 2011. The demographic information for each patient consists of the membership number, gender, birth date, and the visiting dates, where the frequency can be measured by counting the number of visits during the time period. Monetary value for each patient is excluded in this study since the majority of the income from a dental clinic is from the fixed copayment and registration fee per visit by national health insurance program in Taiwan [2–4]. The notations used in this study are as follows. The numbers of 1 and 0 represent males and females, respectively. The birth date is classified into four age groups, that is, 5 years old and below with the number of 1, 6–10 years old with the number of 2, 11–15 years old with the number of 3, and 16 years old and above with the number of 4. The definition of LRFM model is described in Table 1.

The descriptive statistics of length, recency, and frequency are summarized in Table 2. Specifically, if the first and the last visit dates are identical, the length is defined as one. In this study, the maximum and minimum values of length in terms of days are calculated to be 5,377 and 1, respectively. The maximum and minimum recency values are 728 and 9, respectively. The larger the recency value is, the more recent the patient visits. Finally, the maximum and minimum values of the frequency are 19 and 1, respectively. The characteristics of length, recency, and frequency for different genders and age groups are further depicted in Table 3. The number of male patients is slightly higher than the number of female patients. The majority of patients fall in the ages of 6–10 years old.

**Figure 3:**
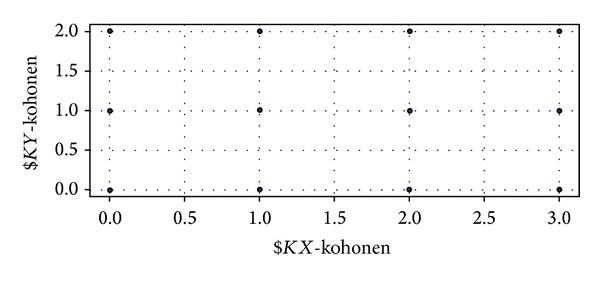
Twelve clusters generated by SOM technique.

To perform cluster analysis, IBM SPSS Modeler 14.2 is the software, and the “Kohonen node” (viz. SOM) with default values is used. In addition, the Kohonen mode is set to “simple” for cluster analysis. The result generated by SOM suggests that the best number of clusters among 1,462 patients is twelve as shown in Figure 3 based on the characteristics of length, recency, and frequency. Later, the number twelve is set up when* K*-means method is performed for cluster analysis.  Table 4 depicts the descriptive statistics of twelve clusters in terms of sample size, average numbers of* L*,* R*, and* F*, average age and gender of the patients, and the symbol(s) of* L*,* R*, and* F* greater than the averages of* L*,* R*, and* F*. Most of the patients are in Clusters 3, 4, 8, 9, 10, and 12. Patients in Clusters 4 and 8 are younger children. More detailed analyses regarding gender and age group are reported in Table 5.

From Table 4, Clusters 2, 6, and 9 have* L*,* R*, and* F* values greater than the average* L*,* R*, and* F* values. Patients in Cluster 8 have larger* R *and* F* values. Clusters 1, 5, and 7 have higher* L* value compared with the average* L* value. Cluster 4 has larger* R* value, while Cluster 11 has larger* F* value. Finally,* L*,* R*, and* F* values in Clusters 3, 10, and 12 are smaller than the average* L*,* R*, and* F* values.

**Figure 4:**
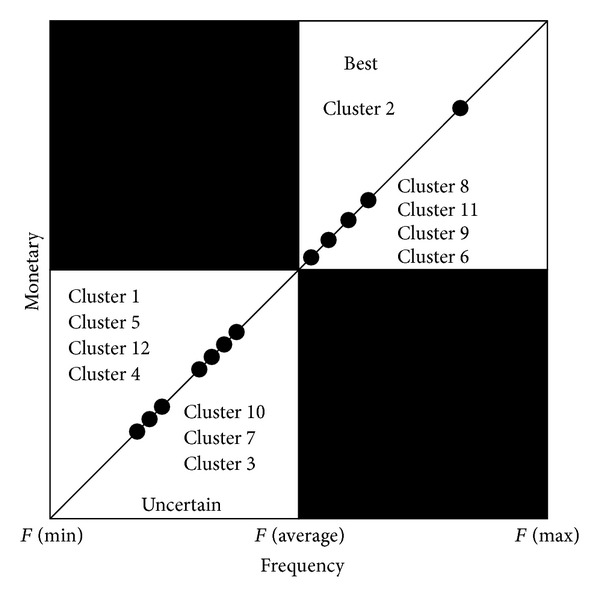
Twelve clusters depicted in customer value matrix.

**Figure 5:**
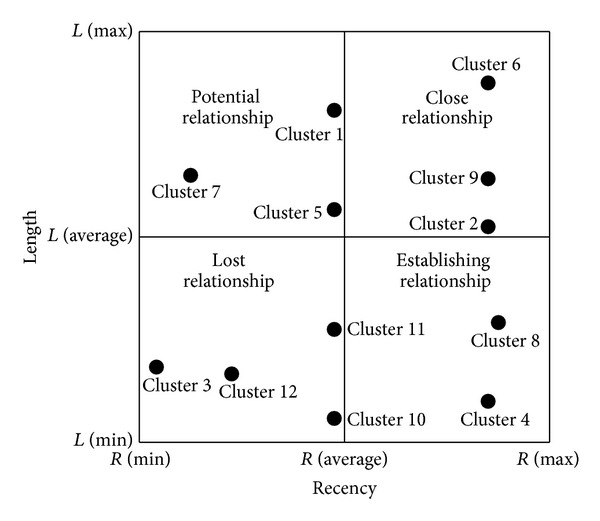
Twelve clusters in customer relationship matrix.

To further analyze patients' values, twelve clusters can be plotted by customer value matrix in terms of frequency and monetary. It is worth noting that monetary is assumed to be fixed in our study such that the original vertical axis (monetary) in Figure 1 can be replaced by frequency. That is, twelve clusters can only be plotted on the straight line through the origin in zones of the best and uncertain customers in Figure 4. Clusters 2, 6, 8, 9, and 11 can be viewed as best customers whereas Clusters 1, 3, 4, 5, 7, 10, and 12 are uncertain customers. In contrast to customer value matrix, customer relationship matrix (Figure 5) shows another scenario in terms of length and recency. Patients in Clusters 2, 6, and 9 belong to close relationship. Patients in Clusters 1, 5, and 7 are the potential relationship. Establishing relationship consists of Clusters 4 and 8. Finally, lost relationship includes Clusters 3, 10, 11, and 12.

## 4. Conclusions

This study uses a two-stage approach, namely, self-organizing maps and* K*-means method, along with LRFM model to cluster a total of 1,462 pediatric patients into twelve groups. From managerial viewpoints, patients in Cluster 11 belong to new customers. In addition to Cluster 11, patients in Cluster 8 are new customers with recent visits. The only difference between Cluster 11 and Cluster 8 is that patients in Cluster 8 have higher frequency to visit the dental clinic. In order to maintain a long-term relationship, the dental clinic needs to interact with the patients more often. In contrast to new customers, patients in Clusters 2, 6, and 9 belong to loyal customers. In order to increase the number of the visits, the dental clinic can provide better after-medical care activities. Finally, patients in Clusters 3, 10, and 12 with low length, recency, and frequency values might not be the focal point for marketing strategy since these patients contribute less to this dental clinic. More resources can be deployed elsewhere to create more profits for this clinic.

From the viewpoints of customer value matrix, Clusters 2, 6, 8, 9, and 11 are the best customers. By directly observing the symbols of* L*,* R*, and* F* in Table 4, Clusters 8 and 11 are viewed as new customers, while customer value matrix considers the patients in Clusters 8 and 11 the best customers. If frequency is a critical factor to analyze patients' values in dental clinic, patients in Clusters 8 and 11 can be taken into account. However, since monetary is assumed to be fixed and replaced by frequency, the revised customer value matrix might not provide accurate information to analyze patients' values. On the other hand, if length and recency are the two critical factors to analyze patients' values, customer relationship matrix shows that Clusters 2, 6, and 9 have close relationship between the patients and the dental clinic. That is, they are loyal patients. Clusters 1, 5, and 7 have the potential relationship with high length but low recency. In other words, these patients can become loyal patients if recency can be increased. In contrast, Clusters 3, 10, 11, and 12 are viewed as lost patients since both length and recency are low. Finally, the dental clinic can try its best to maintain its relationship with patients in Clusters 4 and 8 as time goes by. When the long-term relationship has been established, these patients can become loyal customers in the future.

## Figures and Tables

**Table 1:** The definitions of LRFM model.

Variables	Definitions
Length (*L*)	Refers to the number of days from the first visit date to the last visit date since September 17, 1995
Recency (*R*)	Refers to the number of days since the last visit from July 1, 2009, to June 30, 2011
Frequency (*F*)	Refers to the number of visits in a specified time period (July 1, 2009, to June 30, 2011)
Monetary (*M*)∗	Refers to the copayment and registration fee per visit (∗proposed to be fixed)

**Table 2:** The descriptions of length, recency, and frequency.

	Maximum	Minimum	Average	Standard deviation
Length	5,377	1	1,220.15	1,279.45
Recency	728	9	468.88	219.54
Frequency	19	1	3.55	2.89

**Table 3:** The characteristics of length, recency, and frequency for different genders and age groups.

Variables	Groups	Number	Average length	Average recency	Average frequency
Gender	Male	742	1,223.54	468.93	3.63
	Female	720	1,216.65	468.83	3.46

Age	5 and below	451	282.88	519.77	3.73
	6–10	571	1,019.14	461.86	4.04
	11–15	383	2,254.91	413.76	2.70
	16 and above	57	3,696.75	507.04	2.95

**Table 4:** Descriptive statistics of twelve clusters based on SOM technique.

Cluster	Number of patients	Average length (*L*)	Average recency(*R*)	Average frequency(*F*)	Average age	Average gender	Item (s) above average
1	68	3,543.34	449.34	2.56	13.54	1.49	*L *
2	64	1,372.28	663.73	11.98	6.27	1.44	*LRF *
3	145	416.01	79.24	1.47	7.78	1.50	—
4	236	215.86	655.53	2.19	5.20	1.50	*R *
5	100	1,878.34	438.60	2.43	9.99	1.47	*L *
6	79	4,094.89	655.58	4.08	14.19	1.44	*LRF *
7	107	2,602.78	142.68	1.58	12.30	1.50	*L *
8	170	694.51	673.61	6.67	5.88	1.51	*RF *
9	139	2,257.51	653.85	4.78	10.27	1.52	*LRF *
10	142	136.82	432.74	1.66	6.43	1.51	—
11	71	644.42	457.46	6.06	6.25	1.48	*F *
12	141	403.88	255.55	2.27	7.44	1.49	—
Total	**1,462**	**1,220.15**	**468.88**	**3.55**	**8.17**	**1.49**	

**Table 5:** Distributions of gender and age group for twelve clusters by *K*-means method.

Cluster	1	2	3	4	5	6	7	8	9	10	11	12
Gender												
Male	35	36	72	118	53	44	54	84	67	70	37	72
Female	33	28	73	118	47	35	53	86	72	72	34	69
Age												
5 and below	0	23	44	152	2	0	0	86	2	72	26	44
6–10	4	35	70	62	58	1	22	74	80	47	42	71
11–15	50	6	30	21	36	55	79	9	51	22	3	26
16 and above	14	0	1	1	4	23	6	1	6	1	0	0
Total	**68**	**64**	**145**	**236**	**100**	**79**	**107**	**170**	**139**	**142**	**71**	**141**
